# Die Bright’sche Nierenkrankheit Und Deren Behandlung

**Published:** 1853-03

**Authors:** 


### EDITORIAL DEPARTMENT.

*Die Bright'sche Nierenkrankheit und deren Behandlung. Eine Monographie* von Dr. Fried. Th. Frerichs, ordentlich. Professor der Medicin und Vorstand der Medicinischen Klinik in Kiel. Braunschweig. 1851.

*Bright's Disease and its Treatment; a Monograph.* By Dr. Frederick Th. Frerichs, Professor of Medicine in Ordinary, and Chief of the Medical Clinic at Kiel. Brunswick. 1851.

The history of Bright's disease illustrates, in a very remarkable manner the slow and difficult process by which we attain complete knowledge of a new disease. At first, only the grosser points in its natural history and symptomatology are pointed out; and even these cost the earlier observers much time and labor. Afterward their results are corroborated, or modified, by the labors of others; and the gaps, which were necessarily left in some parts of the structure, gradually filled up. A knowledge of the intimate nature of the malady, and its relation to other diseases, is usually of still more difficult attainment; and many erroneous conclusions must be adopted, many false opinions embraced and rejected, before any one succeeds in establishing a theory of the disease, sufficiently consistent and natural to receive the general assent of the medical public.

Before the publication of Dr. Bright's treatise on diseased kidney, in 1827, the amount of knowledge possessed on this subject was so insignificant, that he must certainly be considered as having explored a new region of pathology; and the disease will probably always be known by his name. Still, the history of the malady was far from being completed by his labors. Even during his time it was already observed that the urine sometimes contained albumen in cases where the autopsies did not show any distinct appearances similar to those of the "granulated kidney;" and some writers attempted to point out the means of distinguishing cases of albuminuria dependent on Bright's disease, from those which had their origin in some other affection. Within a very few years, it was difficult, or impossible, to decide, from the loose and contradictory statements of authors, whether anasarca were necessarily accompanied by albuminous urine, or whether albuminuria always produced anasarca. After the microscope came into vogue, as a means of investigating the minute anatomical alterations of diseased organs, the matter, instead of being simplified, was still farther complicated. Different appearances were seen, not only by different observers, but even by the same observer in different cases; so that some writers, of the highest authority, were led to assume the existence of several different forms of Bright's disease; or rather of several essentially distinct morbid processes, all accompanied by albuminuria. These different affections received names corresponding to the notions entertained by authors as to their nature; fatty degeneration, cirrhosis of the kidney, desquamative nephritis, &c. The opinion, however, has been recently gaining ground that these differences were not owing to any variation in the essential nature of the disease, but depended simply on differences in the *stage*, *intensity*, and *extent* of the morbid process in the diseased organs. It is now well understood that Bright's disease, like many other maladies, may run an acute or a chronic course; that it may attack healthy or cachectic individuals; that it may be moderate or excessive in the degree of its intensity; and that it may invade different portions of the kidney at different periods of time. All these circumstances will, of course, give rise to variations both in the morbid appearances and in the accompanying symptoms, without changing necessarily their essential character and connections. These variations are of so much importance that one of the latest and best writers on the subject, has been led to make use of the expression that "Bright's disease, as a unique and distinctly defined malady, has no existence."

This expression, however, is much too strong. Other diseases present quite as striking variations as this. No one, for instance, will call in question the individuality of Pulmonary Phthisis; and yet the tuberculous deposit may occupy a larger or smaller quantity of the pulmonary tissue: it may be developed slowly or rapidly; it may remain confined to its original limits or extend to neighboring parts; and the whole process, as every observer knows, is subject to constant remittances and exacerbations, and may be permanently arrested, when the deposit is not too abundant, at any one of its different stages. Very much the same thing may be said of Bright's disease; and when these circumstances are taken into consideration, the different appearances, seen by different observers, may be reconciled without difficulty.

The most complete and satisfactory accounts of the views entertained at the present moment, in Europe, on this subject, are to be found in the monograph, by Dr. Frerichs, the title of which is quoted above, and in an article by Dr. Reinhardt, in the annals of the Berlin Charity Hospital, for 1850.

Both writers agree in acknowledging the individuality of the disease, and also as to all the important points of its morbid anatomy, and its relation to other affections. The same opinions are entertained at Vienna by those who are particularly engaged in the pursuit of morbid anatomy in that city.

According to Frerichs, the morbid process which goes on in the kidney may be divided into three stages, of which the first is characterized by congestion and exudation, the second by a metamorphosis of the exuded matter, and the third by its absorption or elimination and the atrophy of the renal tissue. It would, perhaps, be more accurate, strictly speaking, to describe only two stages, viz., the stage of development, in which the congestion and exudation take place, and the stage of retrogression, in which the exuded product becomes altered and finally absorbed. The other division, however, is more practically useful in enabling us to understand the morbid appearances.

In the first stage the kidney is slightly enlarged, and shows, throughout the cortical substance, a strongly marked, diffuse hyperæmia. In consequence of the congestion, an exudation of the fluid parts of the blood takes place everywhere; but more particularly from the contorted capillary vessels of the Malpighian tufts, which project, without any other support than their own walls, into the cavity of the Malpighian capsules. The plasma of the blood is, then, exuded directly into the capsules and the tubuli uriniferi. The albumen and salts pass off with the urine, while the fibrin coagulates in the tubuli under the form of solid, transparent, homogeneous cylinders. If the congestion is very intense, the vessels of the Malpighian tufts (as elsewhere, under similar circumstances) may be ruptured; and then the blood-globules escape also, and may be found suspended in the urine, or entangled in the fibrinous coagula. The coagula themselves, either with or without inclosed blood-globules, are swept out of the tubuli, from time to time, by the fluid which accumulates behind them, and appear as a sediment in the urine. The external surface of the kidney, in this stage, is smooth; and the fibrous capsule is more easily stripped off than usual, owing to a thin sheet of serous fluid which has been exuded beneath it.

From this condition there is a gradual transition to the second stage. The hyperæmia diminishes with the increasing exudation. Not only the tubuli, but the Malpighian capsules themselves, may become filled with fibrinous coagula, and the vascular tufts compressed against their walls. When the exudation is very active, the fibrinous plasma may be deposited, not only in the tubuli and capsules, but also in their interstices; so that the whole organ is considerably swollen, and has an unusually homogeneous aspect on its cut surface.

The second stage is characterized by a fatty degeneration of the epithelium of the tubuli uriniferi, and of the exuded fibrin. The epithelium cells show every grade of transformation, from the deposit of a few fatty granules in their interior, to the stage in which the entire cell is converted into a mass of oily drops and molecules. The cylinders of coagulated fibrin, which were before transparent, or inclosed only healthy epithelium or a few blood-globules, become granulated and finally completely metamorphosed into fatty matter. This transformation is accompanied by corresponding changes in the gross appearances of the kidney. The organ is almost always larger than natural. The dark-red color of the congestive stage has entirely disappeared, and the tissue of the kidney has assumed instead the pale-yellow hue, characteristic of fatty degeneration. When the exudation has been very general, and has taken place into the interstices between the tubuli, as well as into their cavities, it undergoes here, also, the same transformation; and the organ presents on its cut surface a uniform, pale, waxy appearance, which has been often mistaken for an entirely distinct form of disease. Usually, the pyramids retain in a great measure their natural ruddy color, and contrast strongly with the pallid hue of the cortical substance. The fatty matter accumulates in the tubuli and distends them, producing minute roundish *granulations*, which project slightly from the external surface. The texture of the kidney is brittle.

With the third stage commences absorption of the fatty matter. Where the preceding changes have not been so violent or so prolonged as to destroy permanently the functional activity of the tissues, the natural processes recommence as the fat disappears. But where the tubuli have been long obstructed by the fibrinous exudation, where the vessels have been compressed and obliterated, or the blood coagulated in their interior, these parts, also, shrivel and become atrophied, and a general diminution in the size of the kidney results. At the same time the investing capsule becomes closely adherent, and cannot be stripped off without bringing away with it, here and there, small flakes of the cortical substance. As the processes, however, seldom or never go on with the same rapidity in all parts of the organ, those portions which are already atrophied appear as grayish, depressed, fibrous, cicatrix-like furrows, between which are prominences formed by those portions which are still sound, or only in the second stage of the disease. In this way is produced the granulated aspect of "Bright's Kidney;" an appearance altogether different from the more minute granulation of the second stage, which has already been spoken of, and one that can readily be distinguished from it by careful examination. As successive portions of the renal substance pass out of the second stage into the third, the kidney becomes more and more reduced in size, and firmer in consistency; until the cortical substance is reduced in many parts to a condensed, grayish, fibrous layer, not more than a line or two in thickness, and in which no trace of the natural structure of the organ can be distinguished.

From the above description it is easy to see what variations may be met with, both in the gross and microscopic appearances of a kidney affected with Bright's disease. In the first stage we may have every gradation between a moderate hyperæmia, in which the color, consistency and size of the kidney differ but slightly from the natural, and a state in which the organ is turgid, and infiltrated throughout with a dark, bloody fluid. The fibrinous coagula may be abundant or rare, colorless or filled with blood-globules. In the second stage, the organ may show, in different portions, the fatty degeneration confined to the contents of the tubuli, pale-yellow striæ or spots, minute granulations, or the smooth, waxy luster of general fatty infiltration. At all times fresh exacerbations may take place, and active congestion, or even bloody extravasation show itself anew in different localities. Usually the atrophy of the tissues in the third stage will be more or less complete, according to the amount of the previous infiltration.

In the chapter on Special Symptomatology, the nature and origin of *dropsical effusions*, in Bright's disease, are discussed at some length. This symptom is, of course, regarded as one of the most common accompaniments of the disease; but, at the same time, the author is very positive that it is not by any means a constant one. On the contrary he asserts, as Bright had previously done, that the malady "may become perfectly developed, and may even terminate fatally by either acute or chronic processes, without the effusion of a single drop of fluid into the cellular tissue at any period of its course." The dropsy is also liable to the greatest fluctuations in quantity, and changes in position; and may disappear entirely for a time, to return again on the occurrence of any slight exposure. From an examination of 430 cases, reported by himself and others, the author finds that the proportion of cases with dropsical effusion to those without, was about 7 to 1. If the comparison, however, was confined to those cases in which autopsies were made, the proportion was different, viz., about 4 to 1; — a proof, as the author intimates, that the existence of the disease was often overlooked during life. The difference, however, might be as easily explained by supposing the disease to be more generally fatal when unaccompanied by dropsy than when this symptom is present.

As to the origin of dropsy in Bright's disease, the author is clearly of opinion that the common explanation of its occurrence is not satisfactory. It is usually taken for granted that the watery condition of the blood, owing to its loss of albumen, is a sufficient cause of its transudation through the walls of the vessels. It should be remembered, however, that such a state of "hydræmia" may be produced by abundant hæmorrhages, by profuse suppurations, &c., without any dropsical effusion following in consequence. In acute cases of Bright's disease, also, the dropsy often appears simultaneously with the congestion of the kidney, long before the blood has suffered any considerable diminution in the proportion of its solid ingredients. In these cases the dropsy has a common origin with the local affection of the kidney, and appears as its accompaniment, not as its consequence. It is evident, therefore, that although a watery condition of the blood, no doubt, predisposes to dropsical effusion, there is still another cause necessary. This cause is of a very obscure nature, but one which certainly has none the less an actual existence. It is designated by Dr. Frerichs to be a "paralysis of the capillary blood-vessels;"—an expression which is evidently nothing more than the sign of an unknown quantity.

Among the most novel and original ideas in the book, are the opinions advanced by the author on the *poisoning of the blood by urea.* All the older and more recent writers, with only one or two exceptions, agree in considering the peculiar nervous symptoms which so often show themselves in the course of Bright's disease, viz., convulsions, blindness, delirium, coma, &c., as owing to the accumulation in the blood of the excrementitious ingredients of the urine. When the kidney is overloaded with the products of congestion, or when large portions of its substance have been atrophied by a long continuance of the morbid process, its functions must necessarily cease more or less completely. It is well known that entire suppression of the urine invariably produces these nervous disorders in the human subject, and that extirpation of the kidneys in animals is soon followed by a train of similar phenomena. Finally, urea has been repeatedly discovered in the blood of individuals laboring under Bright's disease. Frerichs, however, maintains that the nervous symptoms, in these cases, are not produced by a simple accumulation of urea in the blood, but by the presence of *carbonate of ammonia resulting from its decomposition.*

The grounds on which this opinion is based, may be expressed by the three following propositions:

*First.* — Urea, artificially introduced into the blood, does not produce the symptoms of narcotic poisoning.

*Second.* — Carbonate of ammonia exists in the blood and secretions of animals dying from extirpation of the kidneys.

*Third.* — Carbonate of ammonia, injected into the blood, produces symptoms not to be distinguished from those following suppression of the urine.

The author vouches personally for all these facts. The first, indeed, had been previously asserted by other experimenters. Pure urea, injected into the blood of animals, produced no result beyond a temporary increase in the quantity of urine; and when the kidneys had been previously extirpated, the death of the animal was not perceptibly hastened by the injection. The two latter statements are, so far as we know, peculiar to the author. They were established by the two following series of experiments. In the first series "a solution of from thirty to thirty-five grains of urea was injected into the veins of animals, whose kidneys had been previously extirpated. During the first hours after the operation the animals invariably remained free from all morbid symptoms. After a longer or shorter time (an hour and a quarter to eight hours,) they began to show signs of uneasiness, and vomited the contents of the stomach, which consisted, sometimes, of an acid chyme, sometimes of a yellowish, mucous, alkalescent mass. At the same time the vapor of ammonia was discernible in the breath, and the animals were seized with convulsions, which were alternately suspended and renewed, and gradually gave place to increasing coma with stertorous respiration. Sometimes the convulsions were absent, and the animals remained in a soporose or comatose state from the beginning. After death, which took place from two and a quarter to ten hours after the injection, ammonia was in every instance found in the blood in considerable quantity. The contents of the stomach almost always smelt strongly of ammonia and contained abundance of its carbonate; and ammonia was also distinguishable in the bile and other secretions." "The animals whose veins had been injected with urea remained quiet and comfortable so long as the expired air was free from ammonia. As soon, however, as the breath produced visible fumes with muriatic acid, there was an immediate appearance of those disturbances of the nervous system which are characteristic of a poisoning of the blood by urea."

In the second series of experiments the veins of the animals were injected with a solution of carbonate of ammonia. "Immediately afterward they were seized with convulsions, sometimes of great severity, soon followed by unconsciousness. The respiration was difficult, and the expired air loaded with ammonia. There was choking, and vomiting of bilious matters. The insensibility lasted several hours, and so long as it continued, ammonia was expired from the lungs. Gradually these appearances subsided, and the animals slowly returned to consciousness. If a fresh injection of carbonate of ammonia were made during the state of insensibility, the convulsions and vomiting were renewed, and the urine and fæces were discharged involuntarily."

The author, therefore, concludes that a simple accumulation of urea in the circulation is not sufficient to produce symptoms of narcotic poisoning; but that another condition is also necessary, viz., the presence of a peculiar "ferment" in the blood, which induces the decomposition of the urea and the production of carbonate of ammonia. Precisely what this "ferment" is he does not undertake to say, nor does he pretend to demonstrate its presence directly. He considers, however, that its presence may fairly be inferred from the above facts. Consequently he regards a patient laboring under Bright's disease, whose blood is loaded with urea, as in a condition analogous to that of an animal whose veins have been injected with a quantity of *amygdalin.* Notwithstanding the presence of the foreign substance, the animal may remain unharmed for an indefinite length of time; but the ingestion of a single sweet almond is sufficient, by acting as a ferment, to cause the production of an abundance of hydrocyanic acid and oil of bitter almonds, and destroys life instantaneously.

By this hypothesis we are enabled to explain some facts in relation to the narcotic poisoning in Bright's disease, which have never been clearly understood. In cases of latent or mild character, for example, in which the nervous symptoms declare themselves suddenly, this ferment is supposed to be produced by some occasional disturbance of the system, and to decompose suddenly the urea which had been previously retained in the circulation. The explanation of the manner in which the ferment originates will be best given in the somewhat vague and indefinite language of the author. Accordin to Frerichs, "the complicated series of chemical metamorphoses, which are constantly going on in the blood, require apparently only a slight modification to produce the decomposition of so unstable a compound" as urea. Acute diseases which are accompanied by an abnormal metamorphosis of the blood, readily become the occasion for such a decomposition; so that congestion of the kidneys during scarlatina, or after cholera, typhus, &c., is particularly liable to result in narcotic poisoning. It is well known that the albuminuria and anasarca of pregnant women, symptoms of a true Bright's disease in the kidney, may exist for months during gestation, and yet the convulsions and insensibility, which so often follow, ordinarily take place only at the time of delivery (eclampsia parturientium). Prolonged renal conges tion and an accumulation of urea in the circulation may remain without influence on the nervous system until the process of parturition induces a change in the blood, and the decomposition of the urea. Then come the symptoms of narcotic poisoning.

There certainly seems to be good ground for the opinion that these affections of the nervous system are not simply dependent on the presence of pure urea in the blood; and that some other condition, very possibly its decomposition into carbonate of ammonia, is also necessary. As for Dr. F.'s "ferment," however, that must be regarded for the present as having altogether a hypothetical existence. Such a body may readily be supposed to exist in the blood during the acute exanthemata. Indeed, there are many circumstances which render it highly probable that such is really the case with regard to this class of diseases. Still it must be remembered that complete suppression of the urine (from excessive renal congestion after exposure to cold or from extirpation of the kidneys in animals) is invariably fatal in the same way; and it is not easy to understand why the "ferment" should always happen to be so suddenly produced in the healthy body under these circumstances.

As to the frequency of Bright's disease, Dr. Frerichs agrees with most of the later writers that it is much more common than has been previously supposed; and that *no* disease is so frequently overlooked as this, particularly when it is unaccompanied by dropsical effusion. He establishes completely the identity of the disease under its different forms; acute or chronic, simple or complicated with tuberculosis or cardiac disease, occurring during or after scarlatina, after cholera, typhus, small-pox, and measles, and during pregnancy. He estimates the proportion of recoveries to deaths, in the acute form, as two to one; in the chronic form, as one to eight. J. C. D.

